# Rapid and inexpensive drug susceptibility testing for *Mycobacterium tuberculosis* by a nitrate reductase assay (NRA) from western India

**DOI:** 10.1186/1471-2334-12-S1-P17

**Published:** 2012-05-04

**Authors:** Chanda R Vyawahare, RN Misra, NR Gandham, KM Angadi, Mangala Ghatole, Sarita Kothadia

**Affiliations:** 1 Pad. Dr. D.Y. Patil Hospital & Research Center, Pimpri Pune, Western India; 2 Dr. Vaishampayan Government Medical College Solapur, Western India

## Introduction

Increase in multi-drug resistant (MDR) *Mycobacterium tuberculosis* (MTB) strains is a worrisome trend seen during recent years. Rapid detection of MDR strains is very important to restrict their spread in the population. Gold standard methods for drug susceptibility testing (DST) of MTB are either costly or very slow. Nitrate reductase assay (NRA) is one of the methods for rapid detection of resistance. This technique is based on the capacity of *M. tuberculosis* to reduce nitrate to nitrite. The WHO recommends that the NRA be used as direct test on smear-positive sputum specimen or as an indirect test on *Mycobacterium tuberculosis* isolates grown from conventional solid cultures. We evaluated the performance of NRA as rapid, reliable & inexpensive method for drug-susceptibility testing of *Mycobacterium tuberculosis* against first line antitubercular drugs, Rifampicin (RIF) and Isoniazid (INH).

## Methods

80 strains of *M. tuberculosis* isolated from sputum samples of pulmonary tuberculosis patients were subjected to NRA and absolute concentration method for comparison.

## Results

Out of 80 isolates, 12 strains were resistant to INH & 11 strains were resistant to RIF and 9 strains are resistant to both INH & RIF constituting MDR strains. Sensitivities and specificities were 99%, 98% for RIF and 99%, and 100% for INH by NRA as compared to Absolute Concentration. However median time of obtaining results was shorter using NRA (9-10 days) compared to Absolute Concentration (30-40 days).

## Conclusion

We conclude that NRA has the potential to be a useful tool for rapid DST of *Mycobacterium tuberculosis*.

